# Letter to the Editor Regarding “The Effect of Hippocampal Avoidance Whole Brain Radiotherapy on the Preservation of Long-Term Neurocognitive Function in Non-Small Cell Lung Cancer Patients with Brain Metastasis”

**DOI:** 10.1177/15330338231169599

**Published:** 2023-04-24

**Authors:** Donato Pezzulla, Francesca Maurizi, Ettore Rocchi, Sara Peluso, Luigi De Cicco, Silvia Chiesa, Rossella di Franco

**Affiliations:** 1 Radiation Oncology Unit, 96986 Gemelli Molise Hospital, Università Cattolica del Sacro Cuore, Campobasso, Italy; 2 Radiation Oncology, A.O. Ospedali Riuniti Marche Nord, Pesaro, Italy; 3 Dipartimento Scienze Biomolecolari, Unità Biostatistica e Biomatematica—Università di Urbino, Urbino, Italy; 4 Division of Radiation Oncology, 456041 ASST of Olona Valley, Busto Arsizio, Italy; 5 UOC di Radioterapia Oncologica, Dipartimento Diagnostica per Immagini, Radioterapia Oncologica ed Ematologia, Fondazione Policlinico Universitario A. Gemelli IRCCS, Rome, Italy; 6 Department of Radiation Oncology, Istituto Nazionale Tumori-IRCCS-Fondazione G. Pascale, Naples, Italy

Dear Editor,

We enthusiastically read the article by Wang et al,^1^ where they retrospectively investigated the preservation of long-term neurocognitive function in 47 patients diagnosed with brain metastases of non-small cell lung cancer who underwent hippocampal avoidance whole-brain radiotherapy (HA-WBRT), suggesting how HA-WBRT might have a protective effect.

Reading this interesting paper, we identified an inconsistency: it is mentioned that the results of the study revealed how HA-WBRT might have a protective effect on the long-term neurocognitive function”, and in particular the values of the MMSE and the MoCa score are reiterated (Figures 3 and 4 and Tables 4 and 5). From how the data are reported in Tables 4 and 5 and Figure 4, it would seem that individuals who have conducted hippocampal sparing had lower MMSE and MoCA values than those who have not, in contradiction to the data also illustrated in Figure 3. We would like to ask if we had misread the data or if there was a graphic issue in the tables and figures provided.

In conclusion, this is an important study on the protective effect of HA-WBRT on neurocognitive function. Clarification and eventual correction of the aforementioned contradiction will aid in better understanding the findings of this study.
